# Apoplexy of Crooke cell tumour leading to the diagnosis of severe Cushing disease; a case report

**DOI:** 10.1186/s12902-021-00761-2

**Published:** 2021-05-01

**Authors:** Nipun Lakshitha de Silva, Noel Somasundaram, Roshana Constantine, Himashi Kularatna

**Affiliations:** 1 Diabetes and Endocrine Unit, National Hospital of Sri Lanka, Colombo 10, Sri Lanka; 2 Department of Histopathology, National Hospital of Sri Lanka, Colombo 10, Sri Lanka; 3 Neurosurgical Unit, National Hospital of Sri Lanka, Colombo 10, Sri Lanka

**Keywords:** Apoplexy, Case report, Crooke cell tumour, Cushing

## Abstract

**Background:**

Patients with Crooke cell tumours present with features of Cushing syndrome or mass effect. There are few reports of patients with Crooke cell tumours presenting due to apoplexy. All of them had silent tumours. Patients with Cushing syndrome caused by Crooke cell tumours have not been reported to present with apoplexy.

**Case presentation:**

A 35-year-old female presented with sudden onset headache and visual loss for 1 week. She had secondary amenorrhoea for 10 years. There were features of Cushing syndrome including central obesity, multiple monomorphic acne, dorso-cervical and supraclavicular fat pads, hypertension, proximal weakness, pigmentation and refractory hypokalaemia. She was found to have markedly elevated serum cortisol, central hypothyroidism and hypogonadotropic hypogonadism. There was a mass in the sellar region (4.7 cm × 1.9 cm × 5.3 cm) suggestive of a pituitary tumour extending to the suprasellar region. Imaging showed evidence of bleeding and compression of the optic chiasm. She underwent urgent trans-sphenoidal excision of the tumour. Histology was compatible with a pituitary neuroendocrine tumour. There was margination of ACTH reactivity to the cell periphery and ring like positivity in most of the cells in the cytokeratin stain. Features were in favour of a Crooke cell tumour. After surgery she improved gradually and became eucortisolaemic.

**Conclusions:**

This is a unique presentation of an apoplexy of Crooke cell tumour causing Cushing syndrome. Delayed health seeking behaviour of this patient despite severe Cushing disease could have led to this presentation which has not been reported before.

## Background

Crooke cell tumour is a rare type of pituitary neuroendocrine tumour containing Crooke’s hyaline material in the cytoplasm of more than 50% of the tumour cells [1, 2]. These tumour cells stain positively for adrenocorticotropic hormone (ACTH). They have variable degrees of clinical expression of Cushing syndrome. Silent pituitary tumours were reported in 25–35% of the patients [1, 2]. About 80% of these tumours are macroadenomas. Cavernous sinus invasion and suprasellar extension are seen in more than 70% [1, 2]. These tumours tend to be aggressive even following surgical removal with more than 60% recurrence rate [1].

Pituitary apoplexy is haemorrhagic infarction of a pituitary tumour presenting with headache, visual disturbances and sometimes haemodynamic instability [3]. This complication is mostly seen in patients with non-functioning pituitary neuroendocrine tumours and is rare in Cushing disease [4, 5]. Pituitary apoplexy in Cushing disease is limited to several case reports. Cushing syndrome in most of them was diagnosed before the onset of apoplexy [6]. There are few case reports of apoplexy in Crooke cell tumours, all of them being silent. This is in contrast to the fact that most of the Crooke cell tumours are hormonally active [7–10]. We report a young female with previously undiagnosed Cushing disease caused by Crooke cell tumour presenting due to pituitary apoplexy.

## Case presentation

A 35-year-old Sri Lankan female presented with sudden onset painless visual loss leading to complete blindness in the left eye and partial visual loss in the right eye accompanied by sudden onset bilateral frontal headache for 1 week.

On questioning, multiple features of Cushing syndrome were revealed including recent onset weight gain, darkening of skin, widespread acne and difficulty in getting up from the squatting position over several months. She had not sought any medical advice. There was secondary amenorrhoea for 10 years. During the few medical consultations she had for mild acute illnesses, the possibility of Cushing syndrome had not been considered.

Personal medical and family history was unremarkable. There was no history of exposure to any psychoactive substances or glucocorticoids. She was single; and a preschool teacher.

**Fig. 1:**
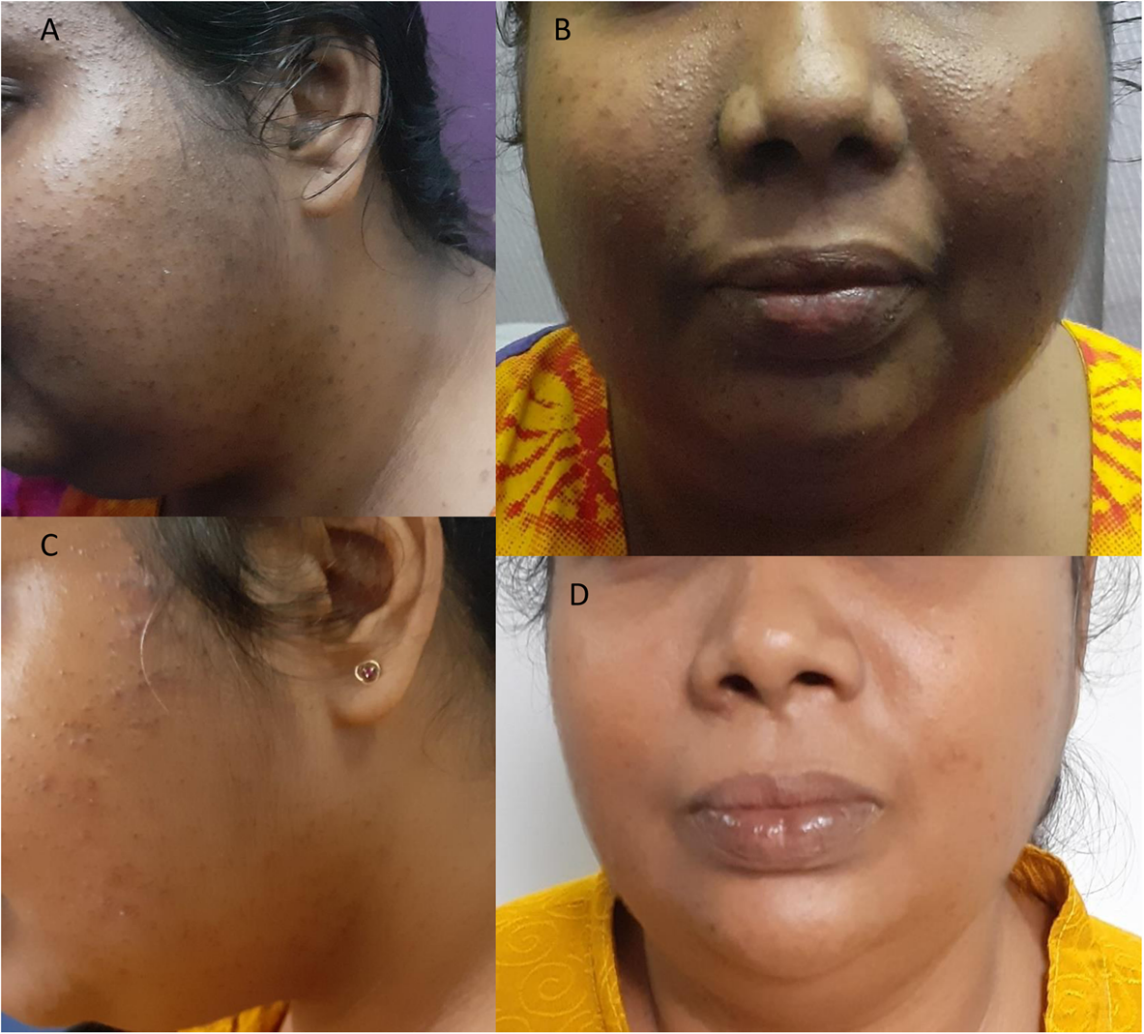
Photographs of the face and upper body taken on initial presentation (**a** and **b**) and four months post-operatively (**c** and **d**). Round face, multiple monomorphic acne over the face and upper chest and dark skin complexion have improved

She was alert and oriented, having central obesity with a weight of 67.6 kg and height of 148 cm. Her Body mass index was 30.86 kg/m^2^. She had pigmentation, multiple monomorphic acne over the face and upper chest (Fig. 1a and b). There were dorso-cervical and supraclavicular fat pads and thin skin over the extremities.

**Fig. 2:**
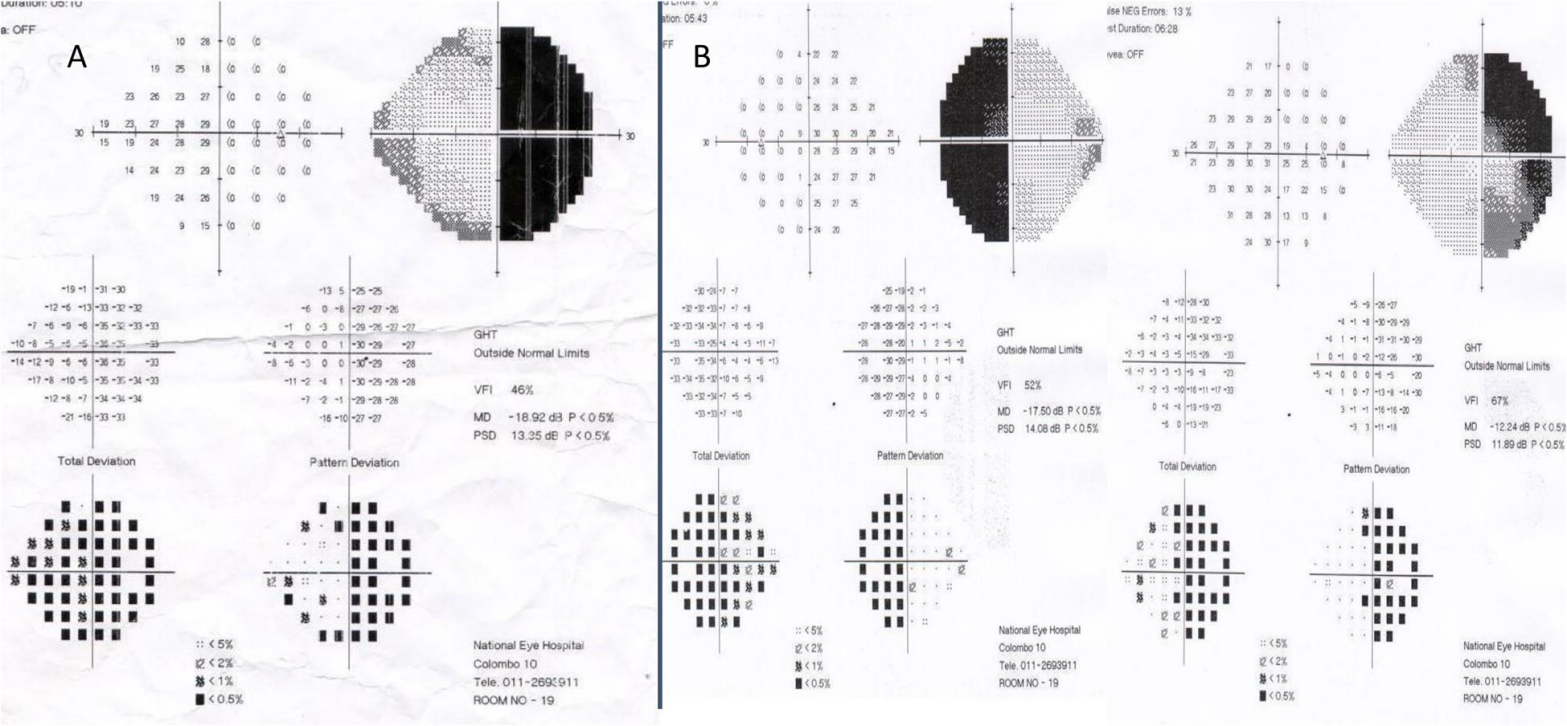
Temporal visual field loss of the right eye is shown in the visual perimetry performed during initial presentation (**a**). Left eye was not tested since there was complete blindness of that eye. Four months after surgery, there is improvement in visual fields with residual bitemporal hemianopia (**b**)

Complete blindness of the left eye and ability to count fingers from the right eye with temporal visual loss were noted (Fig. 2a). The rest of the cranial nerve functions were normal including normal eye movements. She was unable to stand from the squatting position without support. Blood pressure was 150/90 mmHg. The rest of the examination was normal.

Investigations on presentation are summarized in Table 1. Serum potassium remained less than 3 mmol/l persistently despite replacement. There was metabolic alkalosis. Serum cortisol throughout the day was over 1000 nmol/L. Free T4 was 1.04 ng/dL (0.89–1.76) with TSH 0.116 mIU/L (0.5–4.7) whereas LH and FSH levels were low (< 0.007 IU/L and < 0.03 IU/L respectively).

**Table 1:** Summary of Laboratory investigations on presentation

Investigation	Result	Reference range
Haemoglobin (g/dL)	11.6	11–16
White Cell Count (× 10^9^/L)	7.34	4–11
Platelet Count (×10^9^/L)	161	150–450
C- Reactive Protein (mg/L)	< 2	< 6
Serum Creatinine (mg/dL)	0.59	0.5–1.1
Sodium (mmol/L)	141	135–145
Potassium (mmol/L)	2.2	3.5–5.1
24 h urinary potassium excretion (mmol/24 h)	34.56	25–125
Urinalysis	normal	
Aspartate transaminase (U/L)	41	< 40
Alanine transaminase (U/L)	52	< 40
Albumin (g/dL)	3.8	3.5–4.5
Globulin (g/dL)	2.3	2.2–3.5
Bilirubin (mg/dL)	1	0.5–1.1
Fasting blood glucose (mg/dL)	135	< 100
HbA1C (%)	5.8	4–5.7
Arterial blood pH	7.57	7.35–7.45
Arterial Bicarbonate (mmol/L)	35	24–28
Thyroid stimulating hormone (mIU/l)	0.116	0.5–4.7
Free T4 (ng/dl)	1.04	0.89–1.76
9 am cortisol (nmol/l)	1451	118–618
Prolactin (mIU/l)	237	102–496
Follicle stimulating hormone (IU/L)	< 0.3	
Luteinising hormone (IU/L)	< 0.07	
Insulin like growth factor-1 (ng/ml)	87	81–278
Dual-energy X-ray absorptiometry	Bone mineral Density (g/cm^2^) Lumbar spine: 0.814 Right hip: 1.017 Left hip: 0.979	Z-score Lumbar spine: −2.0 Right hip: 0.6 Left hip: 0.4

Cortisol day curve prior to surgery is shown in Table 2.

**Table 2:** Cortisol day curve before and 2 weeks after surgery

Time	Cortisol value (Pre-op) nmol/l	Cortisol value (Post-op) nmol/l
9 am	1451	261
11 am	1094	309
1 pm	1477	300
3 pm	1394	210
5 pm	1358	187

**Fig. 3:**
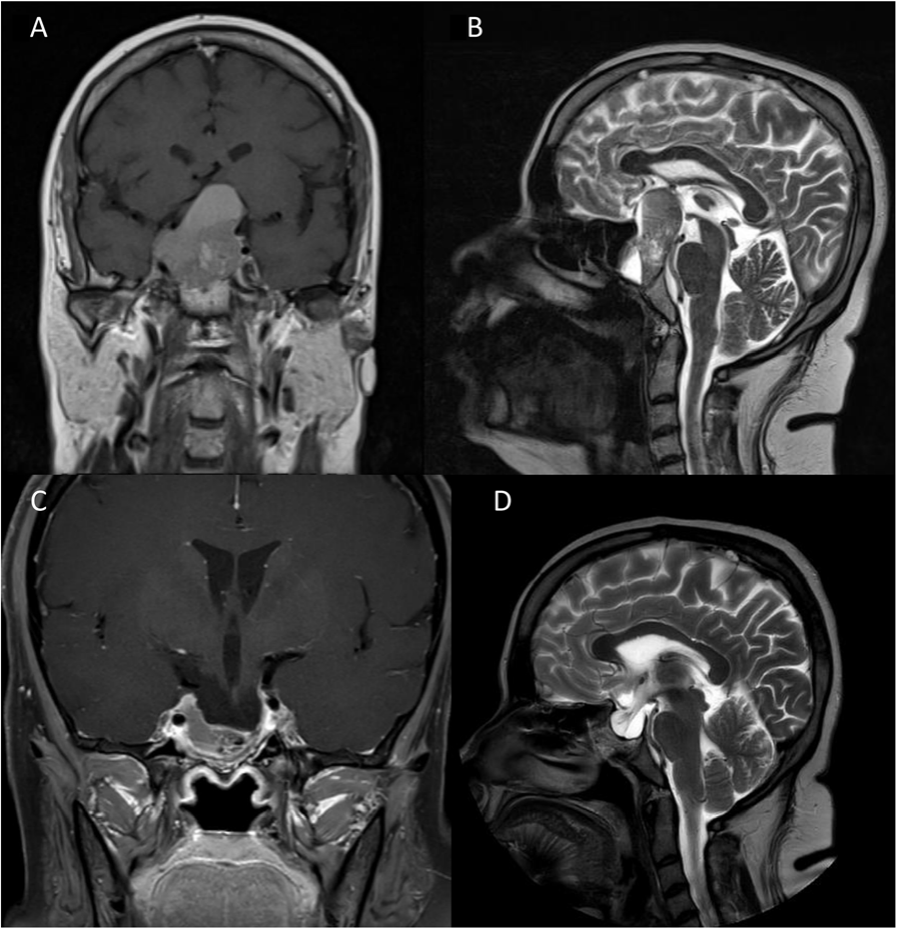
T1 weighted coronal post contrast (**a**) and T2 weighted sagittal (**b**) MRI images before surgery showing large heterogeneously enhancing sellar lesion extending to suprasellar region causing compression of the Optic chiasm and bleeding inside. Displacement of bilateral cavernous sinuses and carotid arteries and compression of third ventricle are seen. T1 weighted coronal post-contrast (**c**) and T2 weighted sagittal (**d**) MRI images of the pituitary region done 3 months after surgery showing no residual tumour in the sellar region. Post-operative changes are noted

**Fig. 4:**
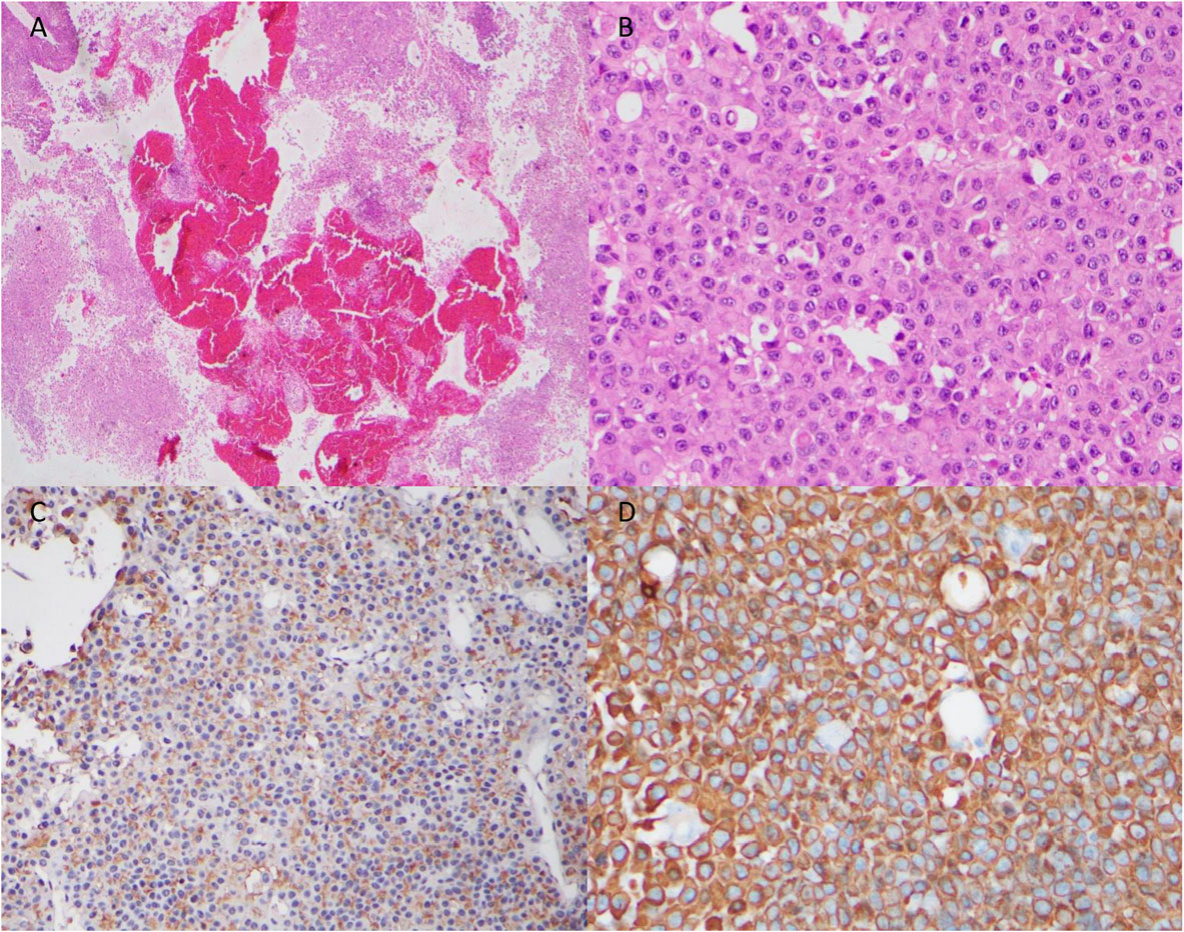
Low power view of H&E staining of surgical specimen showing features suggestive of pituitary neuroendocrine tumour and areas of necrosis and haemorrhage (**a**). High power view of H&E shows cells with basophilic, granular cytoplasm and round nuclei. Some cells showed homogenous pink cytoplasm. **b**. Immunohistochemistry with ACTH staining shows margination of ACTH reactivity to the cell periphery and perinuclear region (**c**). Cytokeratin stain (AE1/AE3) shows diffuse ring like positivity in majority of the cells (**d**)

MRI pituitary showed a heterogeneously enhancing mass in the sellar region (4.7 cm × 1.9 cm × 5.3 cm) extending to the suprasellar region with high T1 fluid level. The pituitary gland was not seen separately (Fig. 3a and b). Features were in favour of a pituitary tumour with bleeding into it. She underwent trans-sphenoidal excision of the tumour 8 days after presentation. Histology was compatible with a pituitary neuroendocrine tumour. On H&E staining, there were cells with basophilic, granular cytoplasm and round nuclei and cells with homogeneous pink cytoplasm. The Ki 67 index was < 1% and p53 staining was 3%. Immunohistochemical staining with ACTH revealed margination of ACTH reactivity to the cell periphery and perinuclear region. The cytokeratin stain (AE1/AE3) showed ring like positivity in most of the cells. These findings were suggestive of a Crooke cell tumour (Fig. 4).

She was started on hydrocortisone and levothyroxine following surgery. Hypokalaemia improved gradually. Her 9 am cortisol after surgery was 261 nmol/L without replacement. Cortisol day curve 2 weeks after surgery without replacement is shown in Table 2.

She improved gradually with regards to pigmentation, acne, facial appearance and proximal weakness (Fig. 1c and d). Blood pressure normalized to 130/80 mmHg. Fasting blood glucose was 98 mg/dl. She regained vision in the left eye with ability to count fingers (Fig. 2b). Three months after surgery, the MRI pituitary showed no evidence of a residual tumour (Fig. 3c and d). Cortisol value in overnight dexamethasone suppression test was < 15 nmol/L. Twenty four hour urinary cortisol excretion was 71.93 μg (55.8–286) and morning cortisol was 181 nmol/L.

Levothyroxine was continued. She was advised to keep stress doses of hydrocortisone. Cyclical oestrogen and progesterone replacement therapy was initiated. The 1 year follow up MRI did not show evidence of tumour recurrence. Twenty four hour urinary free cortisol was 75.07 nmol/24 h (55.5–286). The patient was satisfied with her clinical outcome. The timeline of events are summarised in Table 3.

**Table 3:** Timeline of events with diagnostic tests and interventions

Month/year	Events	Diagnostic tests	Interventions
2009	Secondary amenorrhoea	None	None
From beginning of 2019	weight gain, darkening of skin, multiple acne and difficulty in getting up from squatting position	None	None
August/2019	Sudden onset headache and blindness	Visual assessment: complete blindness of left eye and finger counting from right eye with temporal visual loss MRI-pituitary: giant pituitary tumour with bleeding Biochemistry: hypokalaemia, elevated cortisol, central hypothyroidism, low FSH, LH	Trans-sphenoidal excision of the pituitary tumour Histology: Crooke cell tumour Started levothyroxine and hydrocortisone replacement
November/2019	Follow up	MRI-pituitary: No residual tumour Visual field: bitemporal hemianopia (improved) Twenty four hour urinary cortisol: normal 9 am cortisol: 181 nmol/L	Started cyclical oestrogen and progesterone, levothyroxine continued
September/2020	Follow up	MRI-pituitary: No residual tumour Twenty four hour urinary cortisol: normal	Continued same treatment

## Discussion and conclusions

Apoplexy in patients with Cushing disease is rare and limited to case reports. Patients with Crooke cell tumours present with Cushing disease or due to mass effects when they are hormonally silent. We did not come across any report of a patient with Crooke cell tumour causing Cushing syndrome presenting due to apoplexy. 

There are few case reports of apoplexy in Crooke cell tumours. All of them are in the background of silent tumours. In a 49-year-old female with pituitary apoplexy, subsequent histology was suggestive of Crooke cell tumour. Patient did not have clinical or biochemical evidence of Cushing disease [7]. A 64-year-old male developed apoplexy and did not have any clinical features of Cushing disease. He had a history of a pituitary tumour resection more than a decade before with unknown histology. The recent histology suggested Crooke cell tumour. Preoperative ACTH and cortisol were elevated, but in the setting of acute stress [8].

In a case report of a 45-year-old male, authors describe the interesting finding of apoplexy and two intracranial pseudoaneurysms in a patient with Crooke cell tumour. Clinical and biochemical features were in favour of a silent pituitary adenoma [9]. In another report of a 55-year-old female with apoplexy, endocrine evaluation has suggested a non-functioning pituitary adenoma. It was subsequently diagnosed as a Crooke cell tumour based on histology [10].

Our patient’s clinical picture is quite different from the patients described above. She had obvious features of Cushing syndrome which resolved after surgical resection of the tumour. These included refractory hypokalaemia and multiple discriminatory features such as dorsocervial and supraclavicular fat pads, proximal weakness, multiple monomorphic acne, round face and pigmentation. Therefore this is likely to be the first reported case of apoplexy in a patient with active Cushing disease due to a Crooke cell tumour. This unique presentation could have been partly contributed to by the delayed health seeking behaviour.

There were few limitations we had to face in our setting. We could not arrange ACTH assay or overnight dexamethasone suppression test prior to surgery. She underwent surgery soon after referral to the neurosurgical and endocrine teams as a sight saving measure. We used AE1/AE3 stain as the cytokeratin stain. More specific stain recommended to identify Crooke hyaline is Cam 5.2 [1]. Since this was not available in our setup, we had to depend on the available cytokeratin stain. However in the given context of aggressive pituitary neuroendocrine tumour with lateral and suprasellar extension, peripheral ACTH staining and ring like staining in AE1/AE3 stain, the diagnosis of a Crooke cell tumour could be made.

Our case report brings out a unique presentation of a rare Crooke cell tumour developing apoplexy in the background of progressive Cushing disease. Delayed presentation leading to lack of timely intervention might have resulted in this consequence which has never being reported before.

## Data Availability

The datasets generated and/or analysed during the current study are not publicly available due risk of breach to patient confidentiality, but are available from the corresponding author on reasonable request. Patient’s de-identification will be maintained in sharing the data.

## Abbreviations

ACTH: adrenocorticotropic hormone
MRI: Magnetic resonance imaging
